# Participatory approaches to programme design, planning and early implementation: experiences from a safe surgery project in Nigeria

**DOI:** 10.1093/heapol/czad094

**Published:** 2024-01-31

**Authors:** Kabiru Atta, Jumare Abdulazeez, Farhad Khan, Iyeme Efem, Halimatu Sadiyya Abdullahi, Mansur Dada, Henry C Uro-Chukwu, Karen Levin, Renae Stafford

**Affiliations:** MOMENTUM Safe Surgery in Family Planning and Obstetrics, EngenderHealth, Plot 247 Herbert Macaulay Way, Central Business District, Abuja, Federal Capital Territory, Nigeria; MOMENTUM Safe Surgery in Family Planning and Obstetrics, EngenderHealth, Plot 247 Herbert Macaulay Way, Central Business District, Abuja, Federal Capital Territory, Nigeria; MOMENTUM Safe Surgery in Family Planning and Obstetrics, EngenderHealth, 505 9th Street NW, Suite 601, Washington, DC 20004, United States; MOMENTUM Safe Surgery in Family Planning and Obstetrics, EngenderHealth, 505 9th Street NW, Suite 601, Washington, DC 20004, United States; Independent Consultant, Sokoto State, Nigeria; Bauchi State Health Contributory Management Agency, No. 12 Sarkin Fawa Street, Besides Chartwell Hotel, Bauchi, Nigeria; Department of Community Medicine, Ebonyi State University, Abakaliki, Ebonyi State, Nigeria; MOMENTUM Safe Surgery in Family Planning and Obstetrics, EngenderHealth, 505 9th Street NW, Suite 601, Washington, DC 20004, United States; MOMENTUM Safe Surgery in Family Planning and Obstetrics, EngenderHealth, 505 9th Street NW, Suite 601, Washington, DC 20004, United States

**Keywords:** Programme design, co-creation, surgery, external assistance, development assistance, implementing partner, health systems strengthening, partnerships, agenda, Nigeria

## Abstract

MOMENTUM Safe Surgery in Family Planning and Obstetrics is a global project that strengthens surgical ecosystems through partnership with country institutions. In Nigeria, the project implements in Bauchi, Ebonyi, Kebbi and Sokoto states and the Federal Capital Territory, focusing on surgical obstetrics, holistic fistula care and female genital mutilation/cutting prevention and care. The project utilized participatory approaches during its design, planning and early implementation phases. During the design phase, the project employed a co-creation process featuring a desk review, key informant interviews and stakeholder workshops at community, facility, and government levels to actively listen to, identify and incorporate local perspectives on surgical ecosystem gaps and priorities. Initial findings, shared at state- and national-level workshops, helped collectively identify and prioritize context-specific interventions. The resulting co-created workplan features interventions to strengthen surgical services based on the National Surgical, Obstetrics, Anaesthesia and Nursing Plan (NSOANP). Upon workplan approval, the planning phase involved meeting with each State Ministry of Health (MOH) to prioritize workplan interventions for implementation and to define the finer details needed to drive early implementation processes. Preliminary achievements during early implementation include state commitments to include a costed facility NSOANP in 2023 annual operational plans, mitigation of health facility staffing shortages and review of national fistula and surgical Health Management Information System indicator data flow and advocacy to the Federal MOH resulting in improved fistula data quality and availability. Well-established state and national systems, structures, policies and guidelines enable this programming approach. Since communication between institutional actors is often limited, these approaches necessitate building and maintaining relationships and knowledge-sharing, which requires a significant up-front time investment that must be balanced with donor/partner desires for rapid deliverables. Linking different actors within the health system together through co-creation/co-implementation represents a crucial step in building sustainable country ownership and oversight for surgical ecosystems strengthening interventions.

Key messages

- This article describes a safe surgery project’s experience in using participatory approaches through its design, planning and early implementation phases. Of note, we used a co-creation approach during the design phase in which key informant interviews at the facility, district and state level and workshops at the state and federal levels identified systems gaps and garnered recommendations for interventions to include in the project workplan.
- Through illustrative examples, we demonstrate how donor-funded health system strengthening projects can intentionally listen to and meaningfully incorporate local stakeholders’ voices beyond the donor and lead implementing partner into essential project processes that contribute to improvements in the surgical ecosystem.

## Introduction

**Figure 1.**
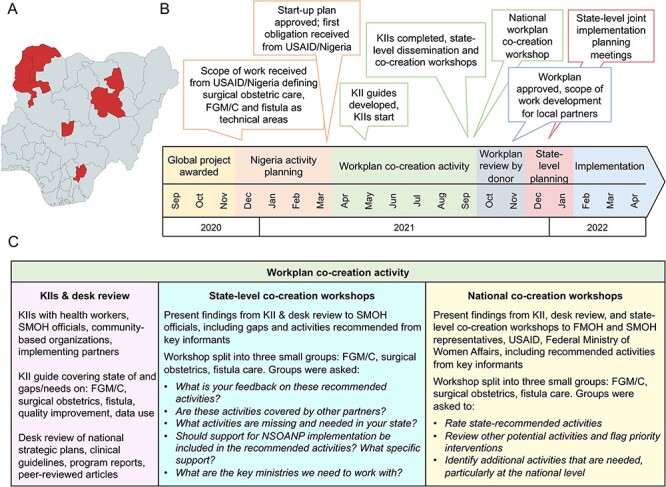
MOMENTUM Safe Surgery in Family Planning and Obstetrics implementation areas, timeline and workplan co-creation activity description

MOMENTUM Safe Surgery in Family Planning and Obstetrics is a global project funded by the United States Agency for International Development (USAID), supporting country institutions to increase access to high-quality, safe and consented surgical care (USAID MOMENTUM, 2021). In Nigeria, the project works to support the provision of safe surgical obstetric care (caesarean section, peripartum hysterectomy), fistula prevention and treatment, and female genital mutilation/cutting (FGM/C) management and prevention by addressing the human resources, processes and enabling environment that comprise the surgical ecosystem (deVries and Rosenberg, 2016). The project implements in Bauchi, Ebonyi, Kebbi and Sokoto states as well as the Federal Capital Territory (FCT) of Nigeria (Figure 1a), covering an estimated population of 24.1 million people (National Bureau of Statistics, 2020). With a high national maternal mortality ratio, 1047 (UI: 793–1565, World Health Organization *et al.*, 2023), low-skilled birth attendance (ranging from 3.4% in Kebbi and 9.2% in Sokoto to 52.1% in FCT), and low caesarean delivery rates (ranging from 0.0% in Kebbi and 0.2% in Sokoto to 8.2% in FCT) in the implementation areas (Nigeria Population Commission, ICF, 2019), limited access to quality surgical obstetric care may be contributing to poor health outcomes. At 53.2%, FGM/C prevalence is particularly high in Ebonyi (Nigeria Population Commission, ICF, 2019). The burden of female genital fistula remains high in Nigeria (Bello *et al.*, 2020), and fistulas originating from iatrogenic causes relating to poorly performed caesarean deliveries represent an emerging and concerning trend (EngenderHealth, 2015; Lawal *et al.*, 2019). Given this reality, targeting development assistance towards safe surgical care may contribute to averting preventable maternal and newborn mortality and morbidity. The project is responding to these challenges by supporting service strengthening at all health system levels, including comprehensive fistula repairs at tertiary-level National Obstetric Fistula Centers (NOFIC), simple fistula repair, caesarean delivery and peripartum hysterectomy at general and secondary-level hospitals, and referral for obstetric emergencies at primary health care centres.

(a) Implementation areas Sokoto, Kebbi, Bauchi, Ebonyi and FCT mapping (map developed on MapChart); (b) start-up/workplan co-creation and implementation timeline; project end date estimated September 2024; (c) workplan co-creation activity description.

The project’s activities in Nigeria are supported and overseen directly through funding from the USAID Nigeria country mission. USAID Nigeria provided a ‘programme description’, a scope of work that prescribes the technical and geographic areas of implementation for this award. This description included fistula care, FGM/C and surgical obstetrics as focal technical areas to be addressed in Bauchi, Ebonyi, Kebbi, Sokoto and FCT.

Following the issuance of a programme description, the implementing partner initiates project design through workplan development. This conventionally takes place primarily between the donor and the lead implementing partner, and may lack alignment with country recipient goals, identified needs and country stakeholder input. The absence of this crucial voice can result in misdirected priorities and activities, potentially jeopardizing country buy-in, ownership, and sustainability. To our knowledge, there are few documented examples or models for how large-scale donor-funded projects can effectively build in stakeholder voices and partnership over the entire course of the project. This article aims to describe one project’s experience using participatory approaches through three of its phases of implementation:

1. **Design**: Co-creation of a project workplan through key informant interviews (KIIs) and workshops in each state and at the federal level.
2. **Planning**: State-specific prioritization of activities from the project workplan through implementation planning meetings in each state.
3. **Early implementation**: Establishing links with state-level institutional actors to jointly plan and implement activities in response to health systems challenges.

## Design, planning and early implementation

Nigeria’s health system is governed by national, state- and local-level institutions (Table 1). The health system is highly decentralized: individual states and FCT manage most health service delivery. At the secondary hospital level, services are managed through State Ministry of Health (SMOH) departments. State Primary Health Care Development Agencies (SPHCDAs) in the local government areas (LGAs) oversee services at primary health care facilities (Table 1, Federal Government of Nigeria). At the state level, both the Honourable Commissioner for Health and the Governor exercise ultimate decision-making authority over health services. As a result of this diffuse governance structure, health systems strengthening programming in Nigeria requires buy-in from multiple actors across various levels of the health system.

**Table 1.** Government of Nigeria institutions implicated in the delivery of safe surgical care at the federal, state and LGA level

Institution/Level	Functions
FMOH	Responsible for the teaching hospitals, specialist hospitals and Federal Medical Centres (including the National Obstetric Fistula Centres) via the Department of Hospital Services Manages the HMIS via the Department of Health Planning, Research and Statistics (DPRS) Provides technical oversight of maternal and newborn health programming, including fistula prevention and care through the Department of Family Health Provides oversight over primary health care services via the National Primary Health Care Development Agency (NPHCDA)
State Ministry of Health (Bauchi, Ebonyi, Kebbi and Sokoto) and Health & Human Services Secretariat (FCT)	Responsible for the general hospitals through the HSMB Manages their State-specific HMIS via the DPRS with technical support from FMOH Provides technical oversight of maternal and newborn health programming, including fistula prevention and care through the Department of Medical Services Provides oversight over primary health care services via the SPHCDA Funds service provision through state/local resources and resource allocations from the federal government
Ministry of Women Affairs (Federal & State)	Manages post-surgical R&R programming for survivors of female genital fistulas Manages empowerment programmes and activities related to adolescents and women, including survivors of GBV for non-medical interventions
SPHCDA and LGA	Manages service delivery at primary health care facilities Validates routine health service data at LGA level and enters data into HMIS and DHIS2

### Design stage: developing a national workplan through KIIs, a desk review, state- and national-level co-creation workshops

The project initiated programme design through co-creation after the donor approved a broad scope of work (Figure 1b). Practitioners, health planners, partner organizations and government officials all participated in a co-creation process involving a desk review, KIIs and state- and national-level workshops to develop a more inclusive and responsive workplan (Figure 1c). A consultant team conducted 120 KIIs with clinicians, SMOH officials and community-based organizations (CBOs) between May and July 2021, inquiring about the state of services and gaps (Figure 1c). We used a purposive sampling approach to target key stakeholders involved in the leadership, management, operations and accountability of the surgical ecosystem. We identified potential national and state level and non-state informants (including CBOs and Civil Society Organizations) based on our existing relationships, our collective knowledge of the health facilities in the implementation areas based on prior projects and our knowledge of potential informants’ involvement in the planning and provision of fistula, FGM/C and surgical obstetric services. KII respondents were asked to identify other stakeholders involved in the planning and provision of such services. The purpose of the KIIs was to approximate the baseline state of the surgical ecosystem, including how services are organized, identifying what gaps exist and soliciting recommendations. For example, KII guides included questions about how conditions are managed, factors essential for preparing facilities for delivering high-quality care, the state of referral systems, data use and quality improvement. Because all the data collected was solely used for the design of a project with a fixed period of performance and we intended to maximize this period for implementation, we did not do subgroup analyses of the interviews by cadre, seniority of post or interview refusals.

A total of five state-level co-creation workshops were held, one in each state and FCT, with SMOH and State Ministry of Women Affairs (MoWA) actors, local non-government organizations, CBOs, CSOs and implementing partners, building off and sharing back KII findings and informant-recommended activities. Each state co-creation workshop was held in the central city for half a day. The workshops included a presentation of the project’s objectives and global theory of change, methods overview and presentation of key findings and recommendations stratified by FGM/C, fistula care and surgical obstetric care. Workshop attendees were then split into three small groups (FGM/C, surgical obstetrics, fistula care) and asked to react to the recommended activities, utilizing discussion questions intended to guide reflection, capture feedback and solicit additional activities in need of support (Figure 1c).

Recommendations from the state-level co-creation workshops were analysed and presented at a subsequent central one-day co-creation workshop, held in Abuja, modelled on the state workshops but with an audience including the Federal Ministry of Health (FMOH), Federal MoWA, state-level government officials, USAID and other implementing partners (Figure 1c). During this workshop, an overview of the project was presented along with the global theory of change, a description of methods including the KIIs, desk review and the state co-creation workshops, and a presentation of key findings, challenges and example state-recommended activities broken down by FGM/C, fistula care and surgical obstetric care. Participants were then split into three small groups: fistula, surgical obstetrics and FGM/C to rate state-recommended activities, review other potential activities and flag priority interventions, and identify additional activities that are needed, particularly at the national level (Figure 1). Workshop feedback was compiled and analysed, and used to draft the project’s workplan, which was submitted to the donor.

Our approach to running the state-level and national-level co-creation workshops differs from, for example, validation meetings following health systems assessments because we attempted to build in opportunities for participants to intentionally add, remove or change recommended activities, particularly during the national workshop. During the state-level workshops, we specifically asked, ‘what is your feedback on these activities?’ and ‘what activities are missing and needed in your state?’ (Figure 1) At the national level, we asked participants to ‘rate state-recommended activities’ and ‘review other potential activities and flag priority interventions’. We split up the workshop audience into small groups specifically to create the conditions to facilitate discussions oriented at prioritizing what interventions should be included in a workplan. This is significantly different from other processes where projects use a start-up workshop to present clearly laid out and developed work plans rather than co-creating workplans with local stakeholders. We did not have an approved workplan in any form prior to KIIs and workshops, in turn enabling participants to materially influence what activities to include in a workplan.

### Planning and early implementation stages: tailoring the national workplan to meet state priorities and collaboration mechanisms for systems strengthening

Following workplan approval, the project held a series of implementation planning meetings with all four states and FCT between December 2021 and January 2022 (Figure 1b). The goal was to tailor the national workplan to state contexts based on which activities were prioritized by SMOH, select LGAs and secondary and tertiary hospital officials; which facilities they wanted to target for project support; and finalize preferred implementation approaches. For example, Sokoto stakeholders expressed a preference for strengthening referral systems for obstetric emergencies whereas stakeholders from Ebonyi prioritized FGM/C prevention and case management. Multiple states prioritized strengthening fistula postoperative rehabilitation and reintegration (R&R) services, while also expressing that support for such interventions required private sector engagement. These meetings are slated to recur annually as the national workplan is updated.

During implementation, approaches for listening, capturing, analysing and integrating local stakeholders’ perspective into activity planning are operationalized. For example, activity concept note templates, which include elements that require project staff to document feedback from key stakeholders, are routinely completed at the start of project activities. Frequent, routine touchpoints with targeted groups of varying actors at different levels of the surgical ecosystem were put in place from weekly to monthly or quarterly depending on what activities are being implemented. For example, the Hospital Services Management Board (HSMB) which is engaged in addressing staffing issues at general hospitals needs to be involved in addressing human resources for health (Table 1). At the facility level, issues that affect the provision or quality of services are discussed between project and facility staff during integrated data review meetings. At the state level, implementing partner meetings and National Surgical Obstetrics, Anaesthesia, and Nursing Plan (NSOANP) platform meetings provide fora to share progress, gauge feedback and identify opportunities for collaboration. Early on, the project established WhatsApp groups in each state including facility medical records officers, facility principal medical officers and state executives from SMOH, HSMB and NOFICs to capture issues in real time and respond accordingly. Project staff brief senior leadership at the SMOH or NOFIC bi- or tri-weekly, though the project has observed that some states are more receptive than others. Lastly, the project relies extensively on informal interactions to link stakeholders together, build buy-in for activities and identify issues.

## Illustrative examples of preliminary achievements and challenges

As the project is early in implementation, the expected achievements of this specific approach at this stage are fundamentally short term in nature. Preliminary achievements to-date include the co-created workplan itself, whose activities align with the NSOANP building blocks (Table 2), as well as examples illustrating how the project is collaborating with government institutions to respond to systems challenges (Table 3). Both of these are needed to strengthen surgical ecosystems as the project continues implementation in the coming years, the former describing the ‘what’ through an ensemble of activities in a workplan and the latter describing the ‘how’.

**Table 2.** MOMENTUM Safe Surgery in Family Planning and Obstetrics fiscal year 2021–22 workplan mapped against implementation states, technical areas and health systems building blocks

	Technical area			Nigeria NSOANP pillar/WHO health systems building blocks					
Workplan activity	Surgical obstetric care	Fistula prevention and treatment	FGM/C prevention and mitigation	Healthcare governance & leadership	Financing of surgical care	Human resources	Infrastructure and equipment	Health information and research	Service delivery
Health facility assessments to identify key gaps	×	×	×			×	×		×
Establishment/strengthening of core interdisciplinary surgical teams at selected health facilities	×	×	×			×			×
Team- and competence-based training for maternity providers on surgical obstetric care	×					×			×
Basic emergency obstetric and newborn care (BEmONC) readiness and referral for comprehensive emergency obstetric and newborn care (CEmONC) services as needed at selected health facilities	×								×
Procurement of vital equipment and consumables for BEmONC and CEmONC facilities	×						×		
BEmONC/CEmONC two-way referral systems from communities to facility and between facilities	×								×
Provider sensitization on maternal health care guidelines for women who have experienced FGM/C			×			×		×	×
Expand availability of holistic fistula care		×							×
R&R services including linkages to auxiliary services (e.g. GBV, social protection)		×							×
Advocacy and community engagement targeting key actors within identified LGAs with high incidence of FGM/C			×						×
FGM/C community sub-committee (with focal points and regular meetings) to ensure linkages between the community, health facilities and social services			×	×					×
Social behaviour change messaging on fistula causes, prevention, treatment and stigma		×							×
Proactive community fistula case finding and facility referral by community volunteers using simple screening tools		×							×
Digital interventions for fistula screening and referral		×							×
State implementation of NSOANP and maternal and newborn health quality improvement frameworks	×	×	×	×	×	×		×	
Dissemination of the National Strategic Framework for the Elimination of Obstetric Fistula, and strategic, costed planning		×		×	×	×			
Capacity building resource mapping (e.g. curricula and job aids) to identify gaps within focus surgical services	×	×	×			×			
Training strategy for medical officers to ensure the delivery of fistula repair as a routine service		×				×			×
Scale-up of catheterization for fistula prevention and non-surgical treatment by midwives		×				×			×
CBO/civil society organization partner capacity strengthening	×	×	×						×
Establishment of state-level fistula data banks to improve data flow, referral and patient-care management		×						×	
Data reporting, review and use for decision-making at supported health facilities	×	×	×					×	×
Develop plans to strengthen HMIS for focus technical areas	×	×	×					×	
Dissemination of knowledge, evidence and best practices through national, regional and global convenings	×	×	×					×	
Qualitative research on informed consent, counselling and postoperative debriefing in surgical obstetric care	×							×	×

**Table 3.** Illustrative examples of collaboration with Government of Nigeria for health systems strengthening

Health systems challenge and implications	Collaboration with Nigerian health systems and structures	Health systems strengthening achievements or short-term mitigations	Next steps for long-term systems strengthening needed or actively pursued
Inadequate staffing at health facilities potentially imperilling NSOANP work plan implementation, as well as the delivery and sustainability of project-supported interventions.	Joint identification of issue and problem-solving during the NSOANP platform meetings in Bauchi, Ebonyi, Sokoto and Kebbi, with SMOH stakeholders (including the Chairpersons of HSMB) indicating inadequate remuneration as the root cause and proposing short- and long-term solutions.	Collaboration with HSMB in affected states resulted in re-allocating health workers between hospitals within each state. Short-term mitigation, whereby the National Youth Services Corps sends doctors to project supported hospitals to fulfill their service year requirements.	Advocacy to ensure laws and policies governing State Health Insurance Scheme and Basic Health Care Provision Fund can support staffing at secondary facilities. Ensure that human resources for health are built into State annual operational plans and budgets, which such funding is released, and increments are secured annually.
DHIS2 with little or no data on fistula, FGM/C and surgical obstetrics from NOFICs and secondary facilities, posing major challenges to data analysis for evidence-based programme planning, budgeting and monitoring, and data for decision making for clinical quality improvement.	Support for FMOH led national-level workshop with all relevant national and state stakeholders to discuss fistula, FGM and surgical obstetric data gaps and to solicit formal data entry permissions to DHIS2 for secondary and tertiary hospitals and NOFICs. Stakeholders include the FMOH Department of Family Health, FMOH Department of Hospital Services, FMOH and SMOH Departments of Planning, Research and Statistics, State HSMB and NOFIC executives.	DHIS2 access granted to secondary health facilities and NOFICs. Facilities to enter routine data directly into DHIS2 and submit hard copies to the LGA monitoring and evaluation officer. Facilities holding data review meetings with all relevant stakeholders and targeted laptop procurement for health facilities to support data entry into DHIS2.	Monitor extent to which facility staffs are entering data into DHIS2 and LGA monitoring and evaluation officers are validating the data monthly, monitor use of data for decision-making at the facility and state levels.
NSOANP not being implemented at the state level, which prevents the integration of surgical, obstetrics, anaesthesia, and nursing care into the health system, risking the coverage and quality of surgical services in the short term and the ownership and sustainability of systems strengthening interventions in the long term.	Establishment of NSOANP platform meetings within all four states and FCT SMOHs, including the Chairpersons of the HSMB. The first objective of the NSOANP meeting was to develop costed facility micro plans.	Dissemination of NSOANP at the State level. Finalization of NSOANP micro plans for the integration of surgical, obstetric, anaesthesia and nursing services in secondary health facilities, integration of the NSOANP into the state health annual operational plan in Ebonyi and Kebbi, and incorporation of costed plans into budgets in Sokoto and Bauchi.	Ensure that the corresponding line items are incorporated into the state budget; ensure funding is released, and that increments are secured annually.
Limited health facility awareness of national policies and strategic plans, risking the implementation of key policies and plans because the needed capacities, structures, systems and action items are left undefined.	Support to NOFIC Bauchi for holding a strategic planning session with management and staff to understand the National Health Act, the National Strategic Framework on the Elimination of Obstetric Fistula and the NSOANP; operationalize these key policies by developing a vision, mission, goals, strategies, and corresponding activities.	Development of a costed 5-year strategic plan for NOFIC Bauchi.	Ensure launch of strategic plan along with resource mobilization plan; mentoring NOFIC Bauchi on strategy implementation.
Resources for post-surgical R&R for fistula care patients are limited, risking gaps in care needed to ensure full recovery.	Support to State Ministries of Health and Ministries of Women Affairs in Kebbi and Sokoto to engage with the private sector for resource mobilization to strengthen R&R services. Support includes capacity strengthening for concept note and budget development; hosting stakeholder meetings to raise consciousness of the need for R&R.	Secure banking sector commitment for financial literacy training and providing access to soft loans to patients receiving fistula treatment. Received consumables for 15 fistula client repairs from the banking sector as part of their corporate social responsibility efforts. Received donated medical equipment, sewing and knitting machines for learning, tailoring machine and a solar-powered water borehole at the rehabilitation centre.	Rehabilitation centre staff capacity strengthening on information and communications technology, financial management, administrative and monitoring and evaluation to ensure that efficient rehabilitation services are provided to fistula patients. Advocate to other states (FCT, Bauchi and Ebonyi) on private sector engagement strategy to attract support for R&R services.
Low coverage of maternal mortality and severe morbidity audits and poor reporting of maternal, child, and perinatal deaths into the national DHIS2 and e-platform at project supported facilities, indicating that death or near miss events are not reviewed and preventative measures are not implemented systematically.	Training facility staff on prior maternal and perinatal death surveillance and response guidelines. Contribution to the FMOH-led development of new MPCDSR guidelines and support for implementation of the new guidelines at project supported facilities.	Increase in the percentage of project supported facilities conducting routine maternal death/near miss audits. Reactivation of MPCDSR committees in supported facilities to perform their roles on maternal and perinatal deaths audit.	Training of facility MPCDSR committees on the new MPCDSR guidelines and tools. Monitor implementation of new MPCDSR guidelines and identify key issues to advocate to state governments for policy and operational change. Strengthen MPCDSR reporting into the DHIS2 and national e-platform.

We experienced a few operational challenges during these early stages. Public health restrictions following a surge in COVID-19 cases delayed KIIs and limited the number of workshops during the design stage. Security issues affected the design and implementation stages, namely in terms of delays and costs incurred. Human resources shortages and turnover of health workers at project-supported facilities may potentially risk the sustainability of implemented activities.

### Surgical ecosystem strengthening activities prioritized in the co-created workplan

The co-created workplan comprises activities that address different challenges of the surgical ecosystem. Most activities support service delivery, such as the establishment of core interdisciplinary surgical teams and team-/competency-based capacity building for surgical obstetric care; others support the state-level implementation of the NSOANP, targeted procurement of equipment, Health Management Information System (HMIS) strengthening and data use and strengthened linkages to R&R services for fistula patients including care and social protection for gender-based violence (GBV) survivors (Table 2). Project design through co-creation yielded interventions that we believe would not have otherwise been included in the workplan absent the process. For example, the inclusion of a fistula data bank (Table 2), a routine health information system that follows fistula patients longitudinally as they are linked to different types of care, specifically arose from inputs of key decision makers who identified the need for these linkages, and would likely not have been otherwise identified as a priority action.

### Illustrative examples of achievements from collaboration on responding to health systems challenges during early implementation

In addition to the implementation of the workplan described above, the project and the SMOH are jointly identifying health systems challenges to surgical service provision, root causes and potential solutions (Table 3). First, the project’s support for state-level implementation of the NSOANP resulted in its incorporation into state annual operational plans, an achievement that will be built on with further advocacy for its incorporation into state annual budgets. Second, to bridge the gap between policy formulation (e.g. the National Strategic Framework on the Elimination of Obstetric Fistula) and on-the-ground health facility implementation, the project supported NOFIC Bauchi to develop a five-year strategic plan to operationalize key health policies and plans, the first of its kind (Table 3). Third, the project also facilitated discussions between hospitals and the various parastatals of the FMOH and SMOH resulting in mutual agreement to provide secondary facilities access to the DHIS2, another step towards institutionalizing the availability and use of routine data for planning, budgeting, monitoring and decision-making for clinical quality improvement (Table 3). Fourth, the project is supporting the SMOH in its attempts at addressing insufficient health facility staffing. Approaches thus far have resulted in short-term mitigation measures, including shifting health workers between facilities and engaging the National Youth Services Corps for doctors (Table 3). As the project progresses, it will advocate the inclusion of human resources for health in state budgets and changes to policies to cover staffing at secondary facilities (Table 3).

## Enablers and constraints

These approaches were primarily enabled by the rich ecosystem of Nigerian governmental and non-governmental institutional actors with deep expertise in the project’s technical areas. For example, while surgical camps are utilized in many parts of the world for fistula repair (Heymann, 2022), Nigeria’s network of NOFICs supports delivery of surgical repair of simple and complex fistulas as a routine health service. CBOs were identified through the co-creation process to lead major project interventions including fistula R&R as well as social behaviour change for fistula, FGM/C prevention and surgical obstetric care. Project funds are provided directly to these CBOs through sub-awards. Several strategic plans, guidelines and policies governing surgical obstetric and fistula care provided the project with pre-established goals to either align with or to advance, including: the NSOANP; the FMOH Guideline on Urethral Catheterization for the Prevention and Management of Obstetric Fistula (project is supporting scale up); the National Protocol on the Management of Complications from FGM (project is supporting health worker sensitization); the National Strategic Framework for the Elimination of Obstetric Fistula; the Reproductive, Maternal, Newborn and Child Health Quality Improvement Framework; and Maternal, Perinatal and Child Death Surveillance and Response (MPCDSR) tools (Nigeria Federal Ministry of Health, 2016, 2018, 2019, 2022). The role of the project can therefore focus on catalysing the implementation of national objectives by addressing bottlenecks in existing systems and structures identified by stakeholders. For example, the project is supporting the implementation of the NSOANP at the state level, as mentioned above, because stakeholders indicated during the design stage’s co-creation workshops that the NSOANP implementation had not been cascaded to the state level.

These activities featured several limitations. First, patients were not included in the KIIs due to ethical constraints; however, CBOs were included that aimed to give voice to community needs. Implementation research studies and project activities that are in progress or anticipated will include inputs directly from patients about experiences of care.

Second, the process was time intensive. Implementing partners and donors interested in applying co-creation to their project design should plan for the time required for coordinating institutional actors. The investment was particularly critical in Nigeria because health system decentralization necessitates consultation with both state- and federal-level government ministries/departments as well as frequent follow-up to ensure necessary approvals from Ministry leadership. Time commitments also increase as the geographic scope of a project increases, partly because of varying receptivity of State actors and political will to external assistance programming, varying State expenditure levels on health as a proportion of total budget, differentiation of programmatic priorities and varying security situations across States. Practically speaking, such investments carry trade-off’s that may delay the delivery of short-term outputs. For example, co-creating the project workplan took six months, delaying implementation start up and support for service strengthening. Our attempt to balance the time commitment did not allow for further subgroup analysis of KII data by health worker cadre or staff seniority, which may have strengthened the reliability and programmatic applicability of the findings but may have also further delayed the start of implementation. Partners interested in adopting this approach may need to balance the required time commitments with the desired level of and the breadth of inquiry. Our formal co-creation process benefitted from explicit donor support of this time-intensive process, and we were transparent about the upfront costs and attempted to manage expectations accordingly.

This up-front time investment adds value over the long term with these approaches representing the first steps in the mainstreaming of surgical ecosystems strengthening interventions into routine government processes. When programme interventions are co-designed and co-implemented with national and subnational stakeholders, the institutional processes and memory needed to implement and sustain them are established, and the measures taken to adapt programming to complex contexts are mutually agreed, implementing partners and state institutions can then effectively begin to plan for mainstreaming into routine government processes. They do this by defining capacity and budgeting benchmarks and timelines for state institutions, identifying a plan for transitioning activities from project to governmental ownership and oversight, and steadily transitioning incrementally over the life of project. As noted in Table 3, for each health systems challenge the project has supported to progress to date, there are highlighted next steps that involve additional government advocacy, and capacity building within country institutions. The extent to which our efforts during these early stages result in longer-term federal and state ownership over project activities indeed remains an open question worthy of evaluation as implementation continues and post-project. However, this experience demonstrates that large-scale donor-funded projects can effectively incorporate local voices, and state and federal inputs in their design, planning and implementation.

## Conclusions

The MOMENTUM Safe Surgery in Family Planning and Obstetrics project is employing a more inclusive process to development assistance programming by routinely listening, collaborating, learning and adapting with national and sub-national stakeholders to explicitly prioritize health interventions in its project workplan and jointly identify and solve surgical ecosystem challenges. These combined efforts aim to strengthen health systems by linking health systems actors to each other to build social capital and trust, as a first step towards long-term local ownership, sustainability and scalability of safe surgical services. Because these approaches are especially time intensive at the start of a project, donors and implementing partners should manage expectations on the delivery of short-term outputs. However, our experience demonstrates that it is possible to meaningfully involve national and subnational stakeholders in the design, planning and early implementation of donor-funded health systems strengthening projects.

## Data Availability

This article describes the experiences implementing a donor-funded health systems project. As such new data were not generated or analysed in support of the development of this article.

## Reflexivity statement

The authors include three females and six males; five are based in Nigeria and four are based in the USA. All authors are global public health development practitioners, and include four clinicians, one technical specialist in fistula programming with expertise in project delivery, and five with expertise in programme monitoring, research, evaluation and learning. Eight of the authors are senior experts with decades of experience supporting health systems strengthening; one author is an early-career professional.
